# Corrosion-Fatigue Life Prediction of the U-Shaped Beam in Urban Rail Transit under a Chloride Attack Environment

**DOI:** 10.3390/ma15175902

**Published:** 2022-08-26

**Authors:** Guixiang Chen, Mingjie Wang, Chenxing Cui, Qingzhang Zhang

**Affiliations:** 1 School of Civil Engineering, Henan University of Technology, Zhengzhou 450001, China; 2 School of Civil Engineering, Central South University, Changsha 410075, China

**Keywords:** chloride attack environment, chloride-induced corrosion, urban rail transit, U-shaped beam, fatigue damage, corrosion-fatigue life

## Abstract

The coupled effect of the chloride attack environment and train load seriously affects the safety and durability of urban rail transit viaducts and dramatically reduces their service life. In this research, a corrosion-fatigue life prediction model of the prestressed concrete (PC) beam under the coupled effect of the chloride attack environment and train load was developed. This proposed model was illustrated by a 30 m-span PC U-shaped beam in an urban rail transit viaduct. The competitive relationship between concrete fatigue cracking time, non-prestressed reinforcement corrosion initiation time, and concrete corrosion-induced cracking time was discussed. The effects of train frequency, the chloride attack environment grade, and the environmental temperature and relative humidity were investigated on corrosion-fatigue life. Results indicate that train frequency, the chloride attack environment grade, and the environmental temperature can reduce the corrosion-fatigue life of a U-shaped beam by up to 30.0%, 50.7%, and 21.5%, respectively. A coupled chloride attack environment and train frequency can reduce the corrosion-fatigue life by up to 61.2%. Distinct from the environmental temperature, the change of relative humidity has little effect on the corrosion-fatigue life of the U-shaped beam.

## 1. Introduction

With the increasing traffic volume on urban roads, traffic congestion is a common phenomenon in the urban city, and the situation is rapidly deteriorating [1]. Urban rail transit has the advantages of large traffic volume, high efficiency, and safety and comfort, which can effectively alleviate the problem of urban traffic congestion and has become one of the leading development directions of modern rail transit [2,3]. During urban rail transit construction, the viaduct has become a common line laying method in urban rail transit due to its benefits of rapid construction, cheaper cost, and excellent line adaptation. The prestressed concrete (PC) beams were extensively employed in the urban rail transit viaduct. As one of the principal structural forms, the urban rail transit viaduct also shows diversified development [4]. Compared to the traditional PC box beam and T-shaped beam, the U-shaped beam is a thin-walled structure with the advantages of reducing the line height, an attractive appearance, excellent soundproof effect, and driving safety that can easily satisfy the comprehensive requirements of the current urban traffic environment [5]. As a result, the PC U-shaped beam has gradually supplanted the traditional PC beam and become a type of beam widely utilized on urban rail transit viaducts [6].

Reinforcement corrosion is widespread in reinforced concrete (RC) structures [7]. The degradation of RC structures is primarily caused by reinforcement corrosion, which is mainly induced by chloride ion intrusion and concrete carbonation [8,9]. However, in coastal areas or deicing salts areas, reinforcement corrosion is mainly caused by the presence of chloride ions [10]. When the RC structures are subjected to the chloride attack environment over an extended period, the chloride ions around the concrete ingress and accumulate on the reinforcement surface. This destroys the reinforcement passive film and eventually causes reinforcement corrosion [11]. Reinforcement corrosion reduces the cross-sectional area of the reinforcement. Corrosion products accumulated at the reinforcement-concrete interface cause the concrete cover to crack and spall, which ultimately reduces the bearing capacity of RC structures [12,13]. The reduction in structural safety and service life caused by reinforcement corrosion results in significant economic losses and cannot be ignored [14]. In addition to the corrosive environment, the RC structure in service also suffers fatigue load, which inevitably causes fatigue damage to the reinforcement and concrete [15]. Fatigue failure of RC structures occurs when fatigue damage accumulates to a limited value in reinforcement and concrete. The fatigue cracking or corrosion-induced cracking of concrete cover accelerates the reinforcement corrosion. Simultaneously, reinforcement corrosion reduces the reinforcement fatigue resistance and accelerates fatigue damage accumulation [16,17]. As a result, the coupled effect of corrosive environment and fatigue loading is more severe than their separation effect or superposition effect, resulting in a noticeable loss of structural performance and a significant reduction in service life.

Currently, some studies have been carried out on the life prediction of RC structures under the coupled effect of corrosive environment and fatigue loading. Cui et al. [18] developed a corrosion-fatigue life prediction model for RC structures under coupled carbonation and repeated loading. The proposed model can predict the corrosion-fatigue life of RC structures simply and conveniently. Sun et al. [19] proposed a corrosion-fatigue life prediction method for RC structures based on fatigue crack propagation and equivalent initial flaw size (EIFS). Zhang et al. [20] introduced a piecewise linear model to simulate the nonlinear behavior of corroded RC beams under fatigue load. The fatigue life can be predicted by simulating concrete and steel strain development under fatigue loading. Bastidas et al. [21] provided a random life prediction and time-varying reliability prediction model for RC beams subjected to the coupled chloride attack environment and fatigue loading. Wu et al. [22] developed the corrosion-fatigue life prediction model for RC beams under the coupled effect of chloride attack and fatigue load based on Fick’s second law and Paris’ law. Recently, the research on U-shaped beams of urban rail transit mainly focuses on static performance [6,23,24] and dynamic performance [25,26,27]. The research on fatigue behavior and corrosion-fatigue life prediction of U-shaped beams is still insufficient. Wang et al. [28] conducted a fatigue test on a full-scale 30 m simply supported U-shaped beam, tested the static performance on the U-shaped beam after the fatigue test, and conducted a finite element analysis. Zhang [29] compiled the fatigue load spectrum under the metro train load and conducted a fatigue test on the full-scale PC U-shaped beam. The results indicate that the cracking occurs at the bottom of the U-shaped beam after 8 × 10^5^ cycles of loading with the fatigue load upper and lower limit values of 240 kN and 25 kN, respectively. Cui et al. [30] established fatigue damage and corrosion-fatigue life prediction model under the coupled effect of carbonation and repeated load. The corrosion-fatigue life of the PC U-shaped beam under coupled carbonation and repeated load were evaluated. In view of the discussions mentioned above, the U-shaped beam of urban rail transit produces irreversible fatigue damage accumulation under the coupled corrosive environment and train load for a long time. However, the corrosion-fatigue life prediction of the U-shaped beam under the coupled effect of chloride attack environment and train load is rarely reported. To maintain the safe operation of urban rail transit, it is critical to assess the corrosion-fatigue life of U-shaped beams under the coupled chloride attack environment and train load.

Based on the operation characteristics of urban rail transit, this study presents a corrosion-fatigue life prediction model of the PC beam for urban rail transit. A 30 m-span PC U-shaped beam on the urban rail transit viaduct in a coastal city was analyzed to illustrate the proposed model. The metro train load model was created to calculate the load impact as the train passes through the beam. The rain flow counting approach was used to calculate the stress range and corresponding cycles of non-prestressed reinforcement in the U-shaped beam. The fatigue damage and corrosion-fatigue life of a U-shaped beam were evaluated under the coupled chloride attack environment and train load. The effects of train frequency, the chloride attack environment grade, and the environmental temperature and relative humidity on fatigue damage and corrosion-fatigue life were discussed. The findings hope to provide insights for the corrosion-fatigue life prediction of PC structures.

## 2. Corrosion-Fatigue Life Prediction Model

### 2.1. Chloride Transport Model

Chloride ions ingress into concrete is a complex process involving several transport mechanisms. The diffusion process is considered the dominant mechanism of chloride ion ingress into concrete. Fick’s second law can be used to explain the diffusion process [31]:

(1) $$\frac{\partial C}{\partial t}=\frac{\partial }{\partial x}\left(D_{\mathrm{cl}}\frac{\partial C}{\partial x}\right)$$

where *C* is the concentration of chloride ion in the environment (kg/m^3^); *D*_cl_ is the diffusion coefficient of chloride ion (m^2^/years); *x* is the distance from the concrete surface (mm); and *t* is the exposure time (years).

When the initial boundary conditions are *C* (*x* = 0, *t* > 0) = *C*_s_ and *C* (*x* > 0, *t* = 0) = *C*_0_, Equation (2) can be obtained as the analytic solution to Equation (1), as follows [32]:

(2) $$C\left(x,t\right)=C_{0}+\left(C_{s}-C_{0}\right)\left[1-\mathrm{erf}\left(\frac{x}{2\sqrt{D_{\mathrm{cl}}t}}\right)\right]$$

where *C*(*x*, *t*) is the concentration of chloride ions in concrete at *x* depth from the concrete surface at exposure time *t* (kg/m^3^); *C*_s_ is the chloride ion concentration around concrete (kg/m^3^); and *C*_0_ is the initial concentration of chloride ion in concrete (kg/m^3^); erf(∙) is the error function.

The corrosion initiation time of tensile reinforcement is affected by many factors, including the initial chloride ion concentration in concrete, chloride ion diffusion rate, concrete cover thickness, and critical chloride ion concentration. When *C*_0_ = 0, *C* (*x*, *t*) = *C*_cr_, and *x* = *c*, the corrosion initiation time *t*_ini_ can be calculated as:

(3) $$t_{\mathrm{ini}}=\frac{c^{2}}{4D_{\mathrm{cl}}}{\left[{\mathrm{erf}}^{-1}\left(1-\frac{C_{\mathrm{cr}}}{C_{s}}\right)\right]}^{-2}$$

where *c* is the concrete cover thickness (mm); and *C*_cr_ is the critical chloride ion concentration of reinforcement corrosion (kg/m^3^).

In addition to the water-cement ratio, the chloride ion diffusion coefficient also depends on the environmental temperature. When the chloride ion diffusion coefficient remains constant over time, *D*_cl_ can be calculated as [33]:

(4) $$D_{\mathrm{cl}}=(7.08w/c-1.846)(0.0447T-0.052)\times 10^{-3}$$

where *T* is the environmental temperature (°C); and *w*/*c* is the water-cement ratio, which can be estimated as [34]:

(5) $$w/c=\frac{27}{f_{\mathrm{cu}}+7.5+13.5}$$

where *f*_cu_ is the cubic compressive strength of concrete (MPa).

### 2.2. Chloride-Induced Reinforcement Corrosion Model

Pitting corrosion occurs in the local position along the length direction of reinforcement in a chloride attack environment. As a result, many corrosion pits are formed on the surface of the reinforcement. Chinese standard GB/T 51355-2019 [33] suggests a non-prestressed reinforcement corrosion model under the chloride attack environment. The corrosion rate of reinforcement is primarily affected by the chloride ion concentration on the reinforcement surface, concrete electrical resistivity, local environmental conditions, and environmental temperature and relative humidity. The corrosion rate *i*_ini_ (mm/year) of reinforcement before the concrete cover cracking can be calculated as:

(6) $$i_{\mathrm{ini}}=11.6\times i\times 10^{-3}$$

where *i* is the reinforcement corrosion current density (μA/cm^2^), which can be computed using the following formulas:

(7) $$\ln i=8.617+0.618\ln C_{s1}-\frac{3034}{T+273}-5\times 10^{-3}p+\ln m_{c1}$$

(8) $$C_{s1}=C_{0}+(C_{s}-C_{0})\left[1-\mathrm{erf}\left(\frac{c\times 10^{-3}}{2\sqrt{D_{\mathrm{cl}}t_{\mathrm{cr},c}}}\right)\right]$$

(9) $$p=k_{\rho }(1.8-C_{\mathrm{cl}})+10{\left(RH-1\right)}^{2}+4$$

where *C*_s1_ is the concentration of chloride ion around the reinforcement (kg/m^3^); *m*_cl_ is the local environmental coefficient (Table 1); *p* is the concrete electrical resistivity (kΩ∙cm); *k*_ρ_ is the coefficient related to the water-cement ratio; *C*_cl_ is the average concentration of chloride ion in concrete cover (kg/m^3^); *RH* is the relative humidity (%); and *t*_cr,c_ is the concrete corrosion-induced cracking time (years).

With the increase in corrosion degree, corrosion products accumulate on the interface between reinforcement and concrete, causing corrosion-induced cracking of concrete cover. At this time, the reinforcement corrosion depth can be estimated as:

(10) $$a_{\mathrm{cr},c}=0.012c/d+0.00084f_{\mathrm{cu}}+0.018$$

where *d* is the initial reinforcement diameter (mm).

The concrete cover cracking increases the paths of chloride ion entry into the concrete [35], resulting in an acceleration of reinforcement corrosion. The time to corrosion-induced cracking of concrete cover can be calculated as:

(11) $$t_{\mathrm{cr},c}=t_{\mathrm{ini}}+\beta_{1}\beta_{2}a_{\mathrm{cr},c}/i_{ini}$$

where *β*_1_ is the correction coefficient for considering the migration of corrosion products around the corrosion pit on the cracking time of concrete cover; *β*_2_ is the correction coefficient for considering multiple corrosion pits distribution on the cracking time of concrete cover.

The concrete cover also cracks when the PC beams are subjected to the train load for a long time. The fatigue cracking time of concrete cover can be calculated by the stress response of concrete at the bottom of the beam, the S-N curve of concrete cracking, and Miner’s rule. The S-N curve of concrete fatigue cracking given in the literature [36] can be expressed as:

(12) $$f_{t}^{\max}/f_{t}=1.3681-0.1214\mathrm{lg}N_{\mathrm{cr}}$$

where $f_{t}^{\max}$ is the maximum tensile stress of concrete under constant-amplitude fatigue load (MPa); *f*_t_ is the concrete tensile strength (MPa); and *N*_cr_ is the number of fatigue loading cycles during which fatigue cracking develops in concrete.

The equivalent maximum tensile stress for variable-amplitude fatigue load can be determined using Miner’s rule. Therefore, the equivalent maximum tensile stress can be calculated as:

(13) $$f_{t,s}^{\max}=\left(1.3681+0.1214\mathrm{lg}\frac{\sum n_{\mathrm{cr},i}\cdot 10^{\frac{f_{t,i}^{\max}/f_{t}-1.3681}{0.1214}}}{\sum n_{\mathrm{cr},i}}\right)\cdot f_{t}$$

where *n*_cr,*i*_ is the number of concrete tensile stress cycles under the different maximum tensile stresses $f_{t,i}^{\max}$; and ∑*n*_cr,*i*_ is the total number of tensile stress cycles in concrete.

The fatigue damage *D*_cr_ of tensile concrete induced by a single fatigue load can be computed as:

(14) $$D_{\mathrm{cr}}=10^{\frac{1.3681-f_{t,s}^{\max}/f_{t}}{0.1214}}\cdot \sum n_{\mathrm{cr},i}$$

When operating at the annual working frequency *f* for *n* years, the cumulative fatigue damage *D*_cr,t_ of concrete can be calculated as:

(15) $$D_{\mathrm{cr},t}=\sum_{i=1}^{n}D_{\mathrm{cr}}f$$

When *D*_cr,t_ reaches 1, the concrete occurs to crack and the corresponding concrete fatigue cracking time *t*_cr,f_ (years) can be obtained.

Concrete fatigue cracking occurs when the maximum tensile stress of the fatigue load on the concrete is greater than the tensile strength of the concrete. The concrete cracking time *t*_cr_ (years) can be taken as the smaller value between the corrosion-induced cracking time *t*_cr,c_ (years) and the fatigue cracking time *t*_cr,f_. Thus, the concrete cracking time *t*_cr_ can be calculated as:

(16) $$t_{\mathrm{cr}}=\min\left\{t_{\mathrm{cr},c},\;t_{\mathrm{cr},f}\right\}$$

When the concrete crack occurs due to fatigue load, the reinforcement corrosion depth *a*_cr,f_ (mm) can be computed as:

(17) $$a_{\mathrm{cr},f}=\left\{ \begin{array}{ll} 0 & t_{\mathrm{cr},f}\leq t_{\mathrm{ini}}\\ i_{\mathrm{ini}}(t_{\mathrm{cr},f}-t_{\mathrm{ini}}) & t_{\mathrm{cr},f}>t_{\mathrm{ini}} \end{array}\right.$$

When concrete cracking occurs (corrosion-induced concrete cracking or corrosion-induced concrete cracking), the corrosion depth *a*_cr_ (mm) can be taken as the smaller value between the corrosion depth *a*_cr,c_ (mm) of corrosion-induced concrete cracking and the corrosion depth *a*_cr,f_ of fatigue cracking. Thus, the corrosion depth *a*_cr_ can be expressed as:

(18) $$a_{\mathrm{cr}}=\min\left\{a_{\mathrm{cr},c},\;a_{\mathrm{cr},f}\right\}$$

Fatigue cracking of concrete also accelerates reinforcement corrosion. It is assumed that the corrosion rate of reinforcement after fatigue cracking and corrosion-induced cracking is equal. The corrosion rate *i*_cr_ (mm/year) of reinforcement after concrete cracking (fatigue cracking or corrosion-induced cracking) can be calculated as [37]:

(19) $$i_{\mathrm{cr}}=(4.5-26i_{\mathrm{ini}})i_{\mathrm{ini}}$$

Then, when the beam operates to the time *t*, the corrosion depth of reinforcement *a*(*t*) can be expressed as:

(20) $$a(t)=\{\begin{array}{ll} 0 & t<t_{\mathrm{ini}}\\ i_{\mathrm{ini}}(t-t_{\mathrm{ini}}) & t_{\mathrm{ini}}<t\leq t_{\mathrm{cr}}\\ a_{\mathrm{cr}}+i_{\mathrm{cr}}(t-t_{\mathrm{cr}}) & t>t_{\mathrm{cr}} \end{array}$$

### 2.3. Corrosion-Fatigue Life Prediction Model

The evaluation method based on the S-N curve of reinforcement is convenient and widely used for fatigue life prediction. The fatigue failure mode of the beam is mainly the fatigue failure of reinforcement [38,39]. The S-N curve represents the relationship between the stress range and fatigue life of reinforcement, which can be expressed as:

(21) $$N=C/{\left(\Delta \sigma \right)}^{m}$$

where *N* is the number of stress cycles at reinforcement fatigue fracture within the reinforcement stress range Δ*σ*; and *C* and *m* are the material coefficient for the reinforcement.

The reinforcement in the beam will produce variable amplitude stress cycles under the train load [40]. The rain flow counting method is used to obtain different stress ranges under variable amplitude stress cycle Δ*σ_i_* and its corresponding number of cycles *n_i_*. The damage caused by the variable amplitude stress cycles of reinforcement can be calculated by Miner’s rule, which can be determined as:

(22) $$D_{b}=\sum \frac{n_{i}}{N_{i}}={\sum n_{i}\left(\Delta \sigma_{i}\right)}^{m}/C$$

where *D*_b_ is the cumulative fatigue damage of the reinforcement, *D*_b_ ≥ 1 indicates the fracture of the reinforcement; *n_i_* is the number of reinforcement stress cycles at the stress range ∆*σ_i_*; and *N_i_* is the number of stress cycles when fatigue failure occurs in the reinforcement under the stress range ∆*σ_i_*.

According to Miner’s rule, an equivalent stress range Δ*σ*_e_ (MPa) with the same damage can also be obtained, and the corresponding number of cycles is ∑*n_i_*, the fatigue failure number *N*_0_ under the action of this equivalent stress range can be obtained according to Equation (21):

(23) $$N_{0}=C/{\left(\Delta \sigma_{e}\right)}^{m}$$

The reinforcement fatigue damage caused by the action of stress range Δ*σ*_e_ is:

(24) $$D_{0}=\frac{\sum n_{i}}{N_{0}}={\sum n_{i}\left(\Delta \sigma_{e}\right)}^{m}/C$$

According to *D*_b_ = *D*_0_, the equivalent stress range Δ*σ*_e_ is expressed as:

(25) $$\Delta \sigma_{e}={\left(\sum \frac{{\left(\Delta \sigma_{i}\right)}^{m}n_{i}}{\sum n_{i}}\right)}^{1/m}$$

The fatigue performance of beams in a chloride attack environment differs from that in a corrosion-free environment. The corroded reinforcement will no longer be suitable for the S-N curve in Equation (21). A coefficient is required for the material constant *C* to indicate its attenuation with time. The time-varying effect of parameter *m* is not addressed in the prediction of corrosion-fatigue life since the attenuation effect of *m* with time is relatively modest. Therefore, the S-N curve in Equation (21) can be rewritten as:

(26) $$N=C(t)/{\left(\Delta \sigma (t)\right)}^{m}$$

The time-variant material constant *C*(*t*) can be expressed as [29]:

(27) $$C(t)=C_{0}\phi (t)$$

where *C*_0_ is the material constant of uncorroded reinforcement; and *ϕ*(*t*) is the attenuation coefficient, which is obtained by fitting the test results of corroded reinforcement in the literature [17,19,41,42,43,44,45,46,47,48,49], and can be calculated as:

(28) ϕ(t)=min{1, −0.0947−0.3659lnw(t)}≥0

where *w*(*t*) is the reinforcement cross-sectional loss ratio, which can be expressed by the ratio of the reinforcement corrosion pit area *A*_pit_(*t*) to the initial reinforcement area *A*_ini_, and the initial reinforcement area *A*_ini_ = π*d*^2^/4.

The reduction in the cross-sectional area increases the stress range of reinforcement, which can be expressed as [50]:

(29) $$\Delta \sigma (t)=\frac{\Delta \sigma_{0}}{\left(1-w(t)\right)}$$

where ∆*σ*_0_ is the uncorroded reinforcement stress range (MPa).

**Figure 1:**
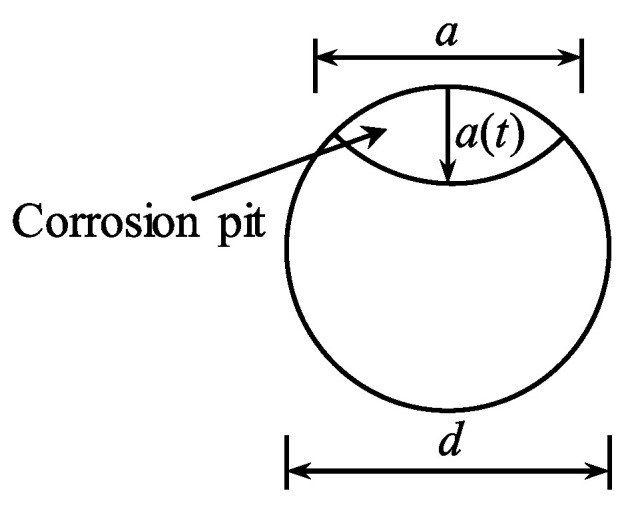
Corrosion pit shape.

The formation and shape of a corrosion pit depend on many factors related to material qualities, manufacturing methods, and electrochemical parameters. For the sake of simplicity, the corrosion pit illustrated in Figure 1 is considered to be spherical [51]. The corrosion pit area can be calculated as:

(30) $$A_{\mathrm{pit}}(t)=\{\begin{array}{ll} A_{1}+A_{2}\; & a(t)\leq \frac{d}{\sqrt{2}}\\ \pi \frac{d^{2}}{4}-A_{1}+A_{2} & \frac{d}{\sqrt{2}}<a(t)\leq d\\ \pi \frac{d^{2}}{4} & a(t)\geq d \end{array}$$

where

$$A_{1}=0.5\left(\theta_{1}{\left(\frac{d}{2}\right)}^{2}-a_{t}|\frac{d}{2}-\frac{{\left(a(t)\right)}^{2}}{d}|\right)$$

$$A_{2}=0.5\left(\theta_{2}{\left(a(t)\right)}^{2}-a_{t}\frac{{\left(a(t)\right)}^{2}}{d}\right)$$

$$a_{t}=2a(t)\sqrt{1-{\left(\frac{a(t)}{d}\right)}^{2}}$$

$$\theta_{1}=2\arcsin\left(\frac{a_{t}}{d}\right)$$

$$\theta_{2}=2\arcsin\left(\frac{a_{t}}{2a(t)}\right)$$

The equivalent stress range Δ*σ*_e_(*t*) of reinforcement can be represented as:

(31) $$\Delta \sigma_{e}(t)={\left(\sum \frac{{\left(\Delta \sigma_{i}(t)\right)}^{m}n_{i}}{\sum n_{i}}\right)}^{1/m}$$

where ∑*n_i_* is the total number of stress range cycles.

The reinforcement fatigue damage *D*_s_(*t*) due to the single train load can be computed as:

(32) $$D_{s}(t)=\frac{{\left(\Delta \sigma_{e}(t)\right)}^{m}\sum n_{i}}{C(t)}$$

The cumulative fatigue damage of steel bars will increase steadily with the extension of service time without taking maintenance measures. When the train operates for *n* years with an annual passing frequency of *f*, the cumulative fatigue damage *D*_c_(*t*) of the beam can be expressed as:

(33) $$D_{c}(t)=\sum_{i=1}^{n}D_{s}(t)f$$

When *D*_c_(*t*) ≥ 1, fatigue failure will occur.

### 2.4. Corrosion-Fatigue Life Prediction Flowchart

**Figure 2:**
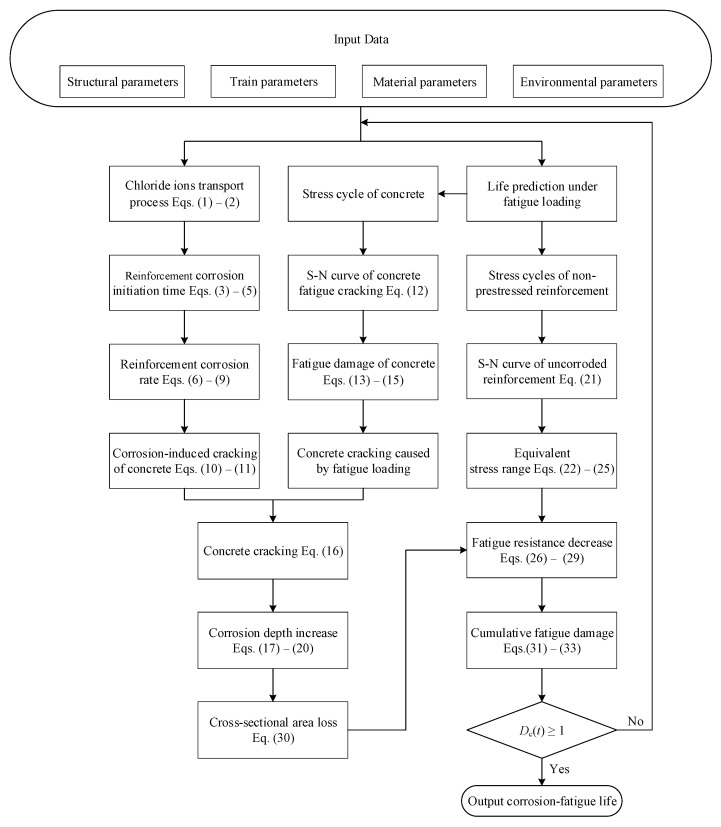
Corrosion-fatigue life prediction process of PC structures.

The above prediction model is applied to a 30 m-span PC U-shaped beam in the urban rail transit viaduct. The flow chart of the calculation process is listed in Figure 2. Additional details of the steps are provided as follows:

(1) Input the structural, train, material, and environmental parameters and calculate the stress range of non-prestressed reinforcement and concrete.
(2) Determine the chloride ion transport process using Equations (1) and (2).
(3) Compute the corrosion initiation time and corrosion rate of reinforcement before the concrete cracking and corrosion-induced cracking time of concrete using Equations (3)–(11).
(4) Calculate the fatigue damage of concrete and concrete cracking time produced by fatigue loading using Equations (12)–(15).
(5) Compute the corrosion rate and corrosion depth following concrete cracking and the concrete cracking time using Equations (16)–(20).
(6) Calculate the cross-sectional area loss of reinforcement using Equation (30).
(7) Obtain the equivalent stress range of uncorroded reinforcement using Equations (21)–(25).
(8) Determine the fatigue resistance reduction behavior using Equations (26)–(29).
(9) Calculate the reinforcement cumulative fatigue damage using Equations (31)–(33).
(10) Output the corrosion-fatigue life by determining if *D*_c_(*t*) reaches 1.

### 2.5. Model Validation

To validate the proposed model, the model is used to predict the fatigue life of corroded RC beams in the literature [46,52]. The predicted results are shown in Table 2. It can be seen from Table 2 that, when compared to the test results of twelve specimens in the literature, the error of seven specimens is less than 7%, demonstrating that the proposed model is reliable. However, there are still five specimens with a large error, which is due to the fatigue test having great discreteness, resulting in a large error between the fatigue life test value and the predicted value of partial specimens.

## 3. Engineering Case Study

### 3.1. Description of U-Shaped Beam and Model Parameters

**Figure 3:**
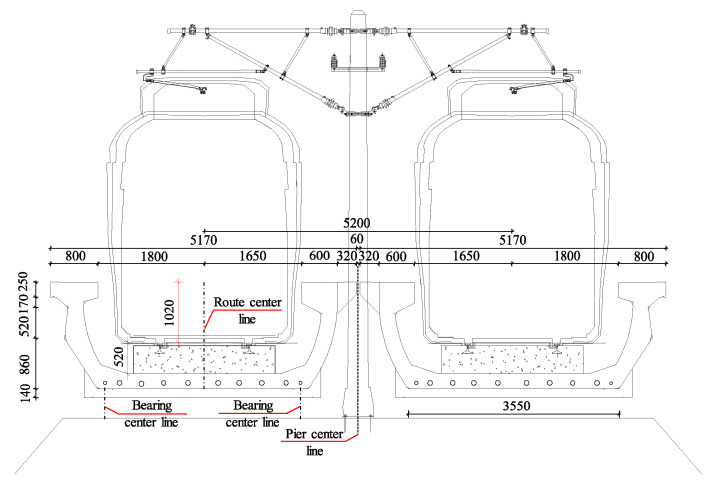
Mid-span section geometry and prestressed reinforcement of the U-shaped beam.

A 30 m-span PC U-shaped beam in an urban rail transit viaduct was evaluated to illustrate the corrosion-fatigue life prediction model of PC structures under the coupled chloride attack environment and train load. The analyzed U-shaped beam with a span of 30 m, depth of 1.8 m, and width of 5.17 m is the most widely used in the whole urban rail transit viaduct. The strength grade of concrete used for the U-shaped beam is C55, and the concrete cover thickness is 35 mm. The non-prestressed reinforcement is HRB400 (a hot-rolled ribbed bar with a yield strength standard value of 400 MPa) with a diameter of 12 mm. Chemical compositions of the non-prestressed reinforcement are listed in Table 3. The prestressed reinforcement is 15.2 mm diameter prestressed strands with the minimum tensile strength of 1860 MPa, and the prestress loss of prestressed reinforcement is calculated by the literature [53]. The design service life of the U-shaped beam is 100 years and the secondary dead load is 35.05 kN/m. The mid-span section geometry and prestressed reinforcement distribution of the U-shaped beam are shown in Figure 3. Table 4 shows the detailed analysis parameters of the U-shaped beam; the parameters of reinforcement and concrete are obtained from the literature [54] and the environmental temperature and relative humidity are obtained from local environmental statistical data.

The urban rail transit viaduct is located on the coast. As a result, the chloride attack environment grade of the U-shaped beam is grade A (Table 5), and the environmental annual average temperature and relative humidity are 15 °C and 65% (Table 4), respectively. The selection of the S-N curve of non-prestressed reinforcement directly affects the final evaluation results when evaluating the corrosion-fatigue life of the U-shaped beam. Song et al. [55] fitted the parameters in the S-N curve of non-prestressed reinforcement in a PC beam. The material constants of *C* and *m* are taken as 1.4213 × 10^10^ and 1.7637, respectively.

### 3.2. Train Load and Frequency

**Figure 4:**
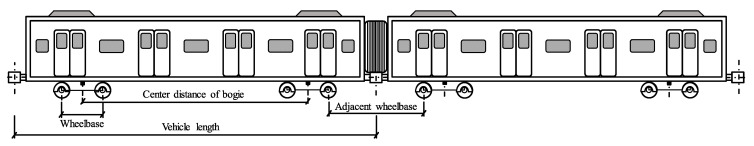
Schematic diagram of B-type metro.

The axle load and wheelbase of metro trains are the main factors affecting the safety performance of bridges. The urban rail transit viaduct operates the B-type metro vehicle, which is a six-carriage train, and the axle weight of the train is 14 t. Figure 4 depicts the axle diagram of the B-type metro vehicle. The B-type metro vehicle has a length of 19 m, a center distance of 12.6 m, a wheelbase of 2.2 m, and an adjacent wheelbase of 4.72 m [56]. When evaluating the corrosion-fatigue life of the U-shaped beam, the train load is simplified as a moving concentrated load passing through the U-shaped beam. The load effect of the midspan section under the moving train load is calculated by the influence line method.

It is necessary to consider the dynamic amplification effect of the moving train load when computing the midspan load response of the U-shaped beam, which can be considered by multiplying the train load by the dynamic coefficient. For the urban rail transit single line, the dynamic coefficient of the U-shaped beam is 1.4 [57]. It is assumed that the train load moves forward every 0.01 m when calculating the load effect of the midspan section. The urban rail transit viaduct operates for 18 h per day. When the average time interval of the metro train is 10, 5, 2.5, and 1.5 min, the train frequencies are 109/day, 217/day, 433/day, and 721/day, respectively.

## 4. Result and Discussion

### 4.1. Fatigue Damage and Corrosion-Fatigue Life

Under the chloride attack environment of grades A to D, the corrosion initiation time of non-prestressed reinforcement in the PC U-shaped beam is 31.7 years, 8.1 years, 5.7 years, and 4.8 years, respectively, and the corrosion-induced cracking time under the corresponding corrosive environment is 41.9 years, 15.8 years, 11.5 years, and 8.6 years, respectively. The fatigue cracking time of the U-shaped beam under four train frequencies of 109/day, 217/day, 433/day, and 721/day are 26.3 years, 13.2 years, 6.6 years, and 3.9 years, respectively.

When the train frequency is 109/day, fatigue cracking of the U-shaped beam develops before the non-prestressed reinforcement corrosion in the A-grade chloride attack environment. Fatigue cracking occurs after corrosion-induced concrete cover cracking in the chloride attack environment of grades B to D. Under the train frequency of 217/day, fatigue cracking of the U-shaped beam occurs before the corrosion of non-prestressed reinforcement in the A-grade chloride attack environment, and fatigue cracking occurs between the non-prestressed reinforcement corrosion and the corrosion-induced concrete cover cracking in the B-grade chloride attack environment. For the chloride attack environment grades of C to D, fatigue cracking occurs following the corrosion-induced concrete cover cracking. Under the train frequency of 433/day, fatigue cracking of the U-shaped beam in the A-grade and B-grade chloride attack environment occurs before the corrosion of non-prestressed reinforcement. The fatigue cracking occurs between the corrosion of non-prestressed reinforcement and the corrosion-induced concrete cover cracking with the C-grade and D-grade chloride attack environment. For the train frequency of 721/day, the fatigue cracking of the U-shaped beam occurs before the corrosion of non-prestressed reinforcement.

**Figure 5:**
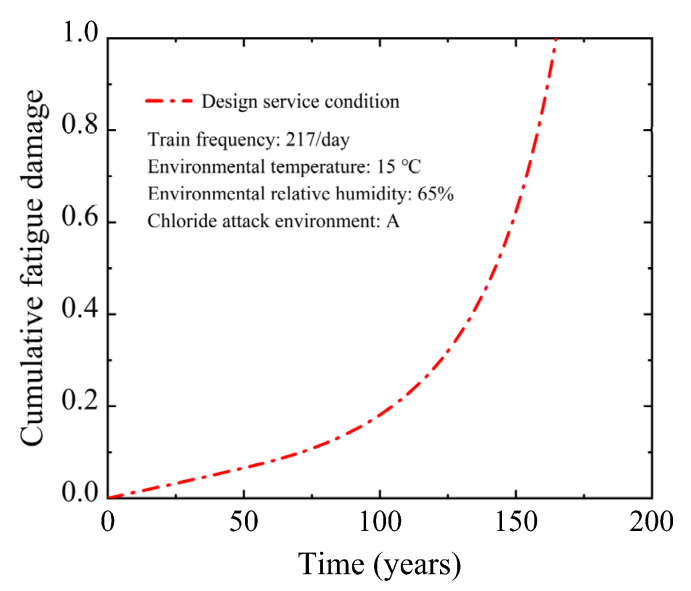
Cumulative fatigue damage of U-shaped beam under design service condition.

Figure 5 depicts the cumulative fatigue damage curve under the design service condition. The cumulative fatigue damage rate of the U-shaped beam grows gradually through the design service life. When the U-shaped beam is operated to the design service life of 100 years, the cumulative fatigue damage in the non-prestressed reinforcement is only 0.181. Continuous corrosion reduced the effective cross-sectional area of the reinforcement, accelerating the accumulation of fatigue damage. Finally, the corrosion-fatigue failure of the U-shaped beam occurred in the 164.8th year. The cracking of the U-shaped beam exposes the reinforcement to the corrosive environments completely, which accelerates the reinforcement corrosion and ultimately affects the safety and durability of the U-shaped beam. Therefore, it is essential to strengthen the maintenance and repair of the U-shaped beam to avoid concrete cracking during the service period.

### 4.2. Effect of Train Frequency and Chloride Attack Environment

The corrosion-fatigue life of the U-shaped beam is impacted by the chloride attack environment grade and train frequency. The corrosion rate of steel bars is affected by the degree of chloride attack in the environment, and the train frequency influences the fatigue damage accumulation rate of concrete and non-prestressed tensile steel bars in the PC beam. To investigate the effects of the chloride attack environment grade and train frequency on corrosion-fatigue life, the cumulative fatigue damage and corrosion-fatigue life of a 30 m-span PC U-shaped beam were evaluated under different chloride attack environments and train frequencies. Under each calculation condition, the environmental temperature and relative humidity are the same as with the design service condition.

**Figure 6:**
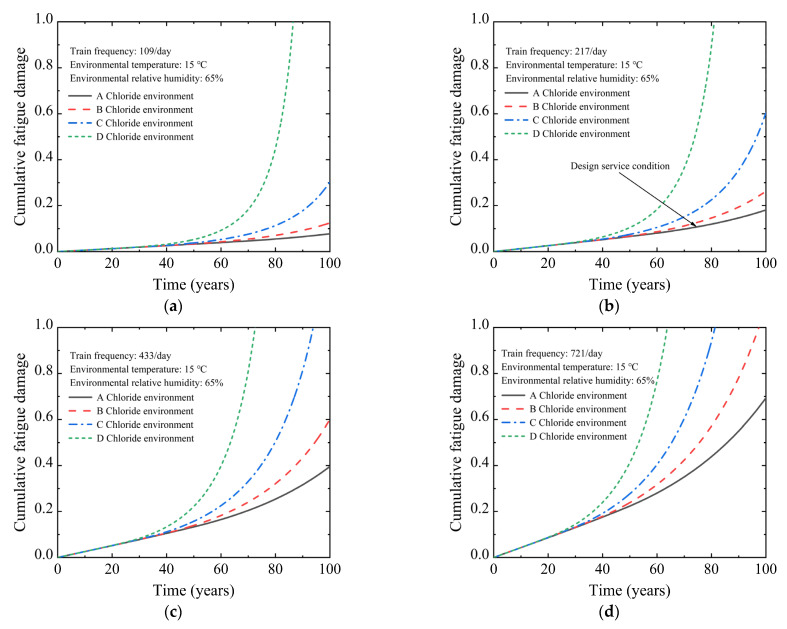
Cumulative fatigue damage of U-shaped beam under different train frequencies: (**a**) *f* = 107/day; (**b**) *f* = 214/day; (**c**) *f* = 433/day; (**d**) *f* = 721/day.

To study the influence of train frequency on the corrosion-fatigue life of the PC beam, Figure 6 displays the cumulative fatigue damage curve of the non-prestressed tensile reinforcement in the 30 m-span PC U-shaped beam under four train frequency grades. As the train frequency rises, the cumulative fatigue damage rate significantly rises. In the case of the U-shaped beam operated to the design service life, the cumulative fatigue damage of train frequencies at 109/day, 217/day, 433/day, and 721/day is 0.078, 0.181, 0.396, and 0.694, respectively, under the A-grade chloride attack environment. Compared with the train frequency of 109/day, the cumulative fatigue damage under the train frequencies of 217/day, 433/day, and 721/day is increased by 132.1%, 407.7%, and 789.7%, respectively. The corrosion-fatigue life of the U-shaped beam decreases continuously with the increased train frequency. Under the A-grade chloride attack environment, when the train frequency increases to 433/day and 721/day, the corrosion-fatigue life is 136.9 years and 115.4 years, respectively. Compared with the train frequency of 217/day, the corrosion-fatigue life is reduced by 16.9% and 30.0%, respectively. When the train frequency is increased to 721/day, the corrosion-fatigue life of the U-shaped beam under the chloride attack environment of grades A to D is 115.4 years, 98.5 years, 81.2 years, and 63.5 years, respectively.

**Figure 7:**
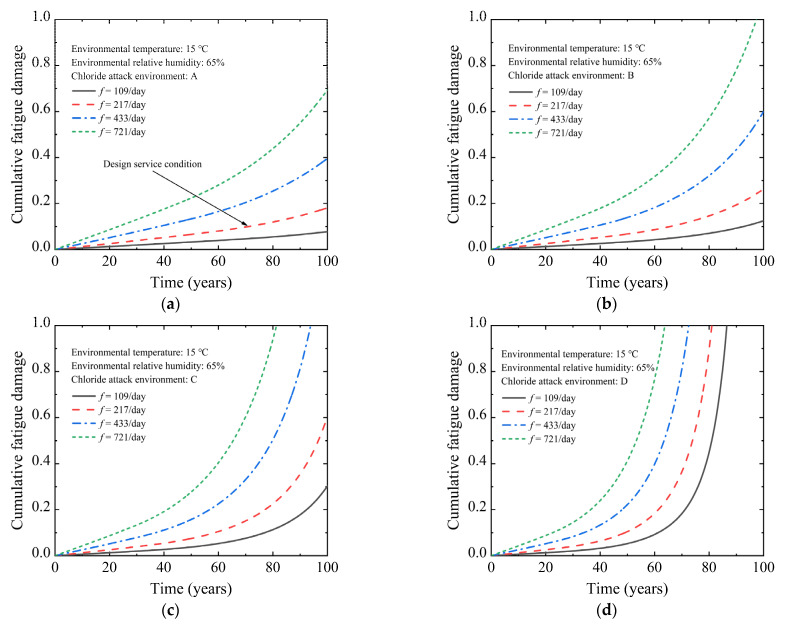
Cumulative fatigue damage of U-shaped beam under different chloride attack environments: (**a**) A chloride attack environment; (**b**) B chloride attack environment; (**c**) C chloride attack environment; (**d**) D chloride attack environment.

Figure 7 depicts the cumulative fatigue damage curve of non-prestressed steel bars in the 30 m-span PC U-shaped beam under four grades of chloride attack environment to evaluate the effect of the chloride attack environment on the corrosion-fatigue life of the PC beam. With the increase in the corrosive environment grade, the corrosion rate increases, which leads to an enormous increase in cumulative fatigue damage. When the U-shaped beam operates to the design service life, the cumulative fatigue damage under chloride attack environment grades A to C is 0.181, 0.263, and 0.603, respectively, under the train frequency of 217/day. Compared to the chloride attack environment of A-grade, the cumulative fatigue damage under the chloride attack environment grades of B and C is increased by 45.3% and 233.1%, respectively. The corrosion fatigue failure of a U-shaped beam occurred in the 81.2th year under the D-grade chloride attack environment due to the cumulative fatigue damage value approaching the ultimate value. The corrosion-fatigue life of the U-shaped beam is greatly affected by the chloride attack environment. Under the train frequency of 217/day, the corrosion-fatigue life is 134.4 years, 107.7 years, and 81.2 years, respectively, as the chloride attack environment grade increases from B to D. Compared with the A-grade chloride attack environment, the corrosion-fatigue life is reduced by 18.4%, 34.6%, and 50.7%, respectively. When the chloride attack environment grade is increased to D-grade, the corrosion-fatigue life of the U-shaped beam at train frequencies of 109/day, 217/day, 433/day, and 721/day are 86.6 years, 81.1 years, 72.7 years, and 63.5 years, respectively.

It can be seen from Figure 6 and Figure 7 that the increase in the chloride attack environment grade and train frequency simultaneously can reduce the corrosion-fatigue life of the U-shaped beam significantly. When the train frequency is increased to 721/day and the chloride attack environment is increased to D-grade, the corrosion-fatigue life is 63.5 years. Compared with the design service life of 164.8 years, the corrosion-fatigue life is reduced by 61.2%.

### 4.3. Effect of Environmental Temperature and Relative Humidity

The environmental temperature and relative humidity of the service environment will affect the corrosion-fatigue life of the PC beam. This research investigates the effects of environmental temperature and relative humidity on the corrosion-fatigue life of a 30 m-span PC U-shaped beam. As a consequence, the corrosion-fatigue life of the U-shaped beam at various environmental temperatures (5–25 °C) and relative humidity (55–85%) was predicted. Under each calculation condition, the chloride attack environment and train frequency are the same as the design service condition.

**Figure 8:**
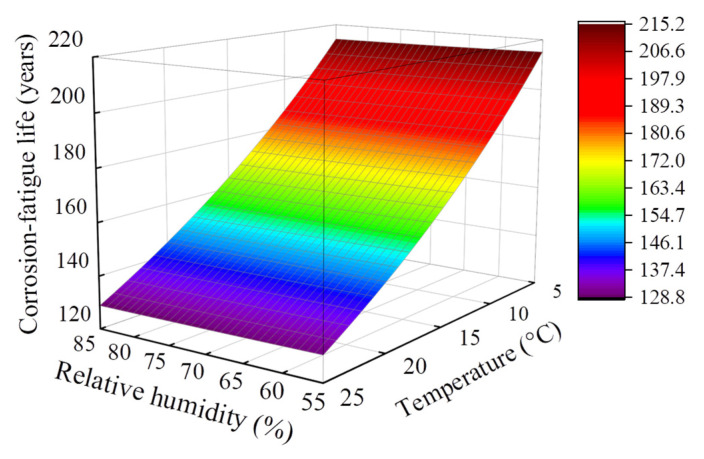
Effect of environmental temperature and relative humidity on corrosion-fatigue life.

As can be seen from Figure 8, when considering the same temperature value, the change of relative humidity will hardly affect the corrosion-fatigue life, which is consistent with the existing study [22]. Unlike relative humidity, the environmental temperature has a more detrimental effect on corrosion-fatigue life. When the environmental temperature is 15 °C, the corrosion-fatigue life is 165.3–164.2 years under the relative humidity of 55–85%. When the relative humidity increases from 55% to 85%, the corrosion-fatigue life of the U-shaped beam decreases by 0.67%. When the relative humidity is 65%, the corrosion-fatigue life is 214.5–129.3 years at the environmental temperature of 5–25 °C. When the environmental temperature increases from 5 °C to 25 °C, the corrosion-fatigue life of the U-shaped beam decreases by 39.7%. Compared with the design service condition, the environmental temperature and relative humidity can reduce the corrosion-fatigue life by up to 21.5% and 0.36%. When the environmental temperature rises to 25 °C and the relative humidity rises to 85%, the corrosion-fatigue life is 128.8 years, which is decreased by 21.9% compared with the design service condition.

## 5. Conclusions

This study developed a corrosion-fatigue life prediction model of PC beam under the coupled chloride attack environment and train load. A 30 m-span PC U-shaped beam in an urban rail transit viaduct was used to illustrate the suggested model. The impacts of train frequency, chloride attack environment grade, and environmental temperature and relative humidity on the corrosion-fatigue life of the U-shaped beam were analyzed. The findings are summarized as follows:

(1) The corrosion-fatigue performance of the 30 m-span PC U-shaped beam is well under the design service condition. After 100 years of operation, the cumulative fatigue damage value is only 0.181, and the final corrosion-fatigue life of the U-shaped beam is 164.8 years. During the operation of the U-shaped beam, it is necessary to strengthen maintenance and repair to avoid concrete cracking.
(2) Increasing the chloride attack environment grade and train frequency decreases the corrosion-fatigue life of the U-shaped beam substantially. The chloride attack environment grade and train frequency, when compared to the design service condition, can decrease the corrosion-fatigue life of the U-shaped beam by up to 50.7% and 30.0%, respectively. The corrosion-fatigue life is shortened by up to 61.2% when the chloride attack environment grade and train frequency increase simultaneously.
(3) The corrosion-fatigue life of the U-shaped beam decreases significantly with the increase in environmental temperature. However, relative humidity has an insignificant effect. The environmental temperature and relative humidity can reduce the corrosion-fatigue life by up to 21.5% and 0.36%, respectively, compared with the design service condition. The increased environmental temperature and relative humidity simultaneously can cause the corrosion-fatigue life to decrease by up to 21.9%.

## Figures and Tables

**Table 1:** Local environmental coefficient.

Local Environmental	*m*_c1_
Non wet/dry alternate environment	2.0–2.5
Hot-humid zone of China	4.0–4.5
Outdoor wet/dry alternate environment and splash zone	4.5–5.5

**Table 2:** Predicted results of corroded RC beams in the literature.

Cross-Section Loss Rate (%)	Actual Stress Range (MPa)	Fatigue Life (×10^4^ cycles)	Predicted Life (×10^4^ cycles)	Reference
26.21	300.00	15.72	24.02	Li et al. [47]
26.84	300.00	22.03	23.50	
26.21	270.00	30.50	28.93	
26.53	240.00	34.70	35.21	
26.84	240.00	36.33	34.83	
26.06	210.00	156.63	45.31	
4.99	210.60	115.90	113.70	Li [53]
8.63	213.80	93.65	88.57	
9.10	210.20	93.08	89.05	
13.68	233.60	41.42	59.83	
19.42	239.90	39.01	45.53	
26.20	246.80	22.55	33.91	

**Table 3:** Chemical compositions of the non-prestressed reinforcement.

Rebar Type	Diameter	Strength Level	Chemical Composition (Mass Fraction (%), Not Greater Than)					
			C	Si	Mn	P	S	Ceq
Non-prestressed rebar	12 mm	HRB400	0.25	0.80	1.60	0.045	0.045	0.54

Note: Ceq = C + Mn/6 + (Cr + V + Mo)/5 + (Cu + Ni)/15.

**Table 4:** Material parameter and environmental factor of the U-shaped beam.

Parameter	Value	Description
*f*_cu_	55 MPa	Cubic compressive strength of concrete
*f*_t_	2.74 MPa	Tensile strength of concrete
*E*_c_	3.55 × 10^4^ MPa	Elastic modulus of concrete
*f*_y_	400 MPa	Yield strength of non-prestressed reinforcement
*E*_s_	2 × 10^5^ MPa	Elastic modulus of non-prestressed reinforcement
*d*	12 mm	Diameter of non-prestressed reinforcement
*c*	35 mm	Concrete cover thickness
*Φ*	15.2 mm	Diameter of prestressed reinforcement
*E*_p_	1.95 × 10^5^ MPa	Elastic modulus of prestressed reinforcement
*f*_p_	1080 MPa	Yield strength of prestressed reinforcement
*C*_s_	2.57 kg/m^3^	A-grade chloride attack environment
*T*	15 °C	Environmental temperature
*RH*	65%	Environmental relative humidity

**Table 5:** Chloride attack environment grade.

Chloride Attack Environment Grade	Environment Type	Chloride Ion Concentration *C*_s_ (kg/m^3^)
A	Offshore atmospheric area (0.5 km from the coast)	2.57
B	Offshore atmospheric area (0.25 km from the coast)	3.83
C	Offshore atmospheric area (0.1 km from the coast)	5.87
D	Atmospheric salt spray area	11.5

## Data Availability

Not applicable.
